# Inverse Langmuir method for oligonucleotide microarray analysis

**DOI:** 10.1186/1471-2105-10-64

**Published:** 2009-02-20

**Authors:** Geert CWM Mulders, Gerard T Barkema, Enrico Carlon

**Affiliations:** 1 Institute for Theoretical Physics, Utrecht University, Leuvenlaan 4, 3584 CE Utrecht, The Netherlands; 2 Institute for Theoretical Physics, Katholieke Universiteit Leuven, Celestijnenlaan 200D, B-3000 Leuven, Belgium

## Abstract

**Background:**

An algorithm for the analysis of Affymetrix Genechips is presented. This algorithm, referred to as the Inverse Langmuir Method (ILM), estimates the binding of transcripts to complementary probes using DNA/RNA hybridization free energies, and the hybridization between partially complementary transcripts in solution using RNA/RNA free energies. The balance between these two competing reactions allows for the translation of background-subtracted intensities into transcript concentrations.

**Results:**

To validate the ILM, it is applied to publicly available microarray data from a multi-lab comparison study. Here, microarray experiments are performed on samples which deviate only in few genes. The log_2 _fold change between these two samples, as obtained from RT-PCR experiments, agrees well with the log_2 _fold change as obtained with the ILM, indicating that the ILM determines changes in the expression level accurately. We also show that the ILM allows for the identification of outlying probes, as it yields independent concentration estimates per probe.

**Conclusion:**

The ILM is robust and offers an interesting alternative to purely statistical algorithms for microarray data analysis.

## Background

Thanks to DNA microarrays the gene expression profiling can nowadays be extended to a genome-wide analysis. Since the first prototypes of the mid nineties [1], microarray technology has advanced considerably in terms of reproducibility and cross-platforms agreement [2]. Quite some effort was also devoted to the development of data analysis tools, which process the raw experimental fluorescence intensities through background subtraction, normalization and eventually summarization. Different microarray platforms, for instance two-color versus single-color arrays, require also different data processing. Most of the current algorithms for DNA microarrays data analysis [3] rely on complex statistical transformations for the above mentioned preprocessing steps. Microscopically based methods [4-6] offer an interesting alternative to purely statistical approaches. These methods use estimates of physical quantities involved in the underlying microscopic processes, as for instance the hybridization free energy, which measures the transcript-probe affinity. The fluorescent intensities are then linked to gene expression levels by means of thermodynamic functions. Input from physics and chemistry is expected to offer a simpler, but still accurate handling of the experimental data.

Here we describe a thermodynamic approach for the calculation of gene expression levels from microarray data, which will be referred to as the Inverse Langmuir Method (ILM). As will be shown below, the ILM determines changes in the expression level accurately, using a simple computational scheme involving a minimal number of adjustable parameters. In Affymetrix Genechips [7] transcripts are interrogated by oligo sequences (25-mers), which are referred to as the probes. The collection of 10 to 20 probes complementary to the same transcript forms a probe set. The ILM allows for the identification of "outlying" probes, for instance due to a faulty genomic annotation, or a high sensitivity for cross-hybridization. The ILM thus also provides feedback for the improvement of the microarray design.

## Results and discussion

To assess the quality of the ILM, we use in this paper the publicly available data from Gene Expression Omnibus with number GSE2521. These data originate from a study [2] of a multi-laboratory comparison of hybridization of the same mRNA sample on three different platforms: Affymetrix oligo, two-color cDNA and two-color oligo arrays. Using mixtures of knockout human cell lines two samples were created in which the expression of few genes is expected to be altered. The study [2] focuses on 16 genes whose expression level was measured by RT-PCR in both samples, in order to compare the log-fold changes with those obtained from Microarray data analysis. Here we are concerned only with Affymetrix data, which were produced by five different laboratories, with two technical replicates each.

In a microarray hybridization experiment several different types of chemical reactions take place simultaneously. A transcript sequence in solution does not only bind to its complementary probe, but may be involved in, for instance, self-folding, it may bind to other partially complementary transcripts in solution, or to non-complementary probes. A review of the physical chemistry of these processes can be found in Ref. [8]. The ILM takes into account a subset of these processes. Nevertheless, a comparison with RT-PCR data shows that it is an accurate method for the estimation of the fold changes of the transcript concentrations.

**Figure 1:**
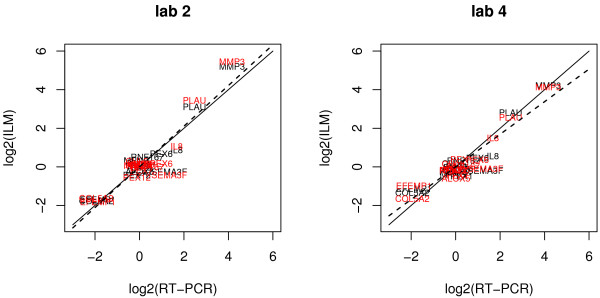
**ILM vs. RT-PCR log-fold changes**. Plot of log_2 _fold changes obtained from the ILM as a function of RT-PCR log_2 _fold changes. The genes are those considered in Ref. [2] and appear with their symbols in the graph. The solid line shown is the diagonal implying perfect agreement between the two estimates. The dashed line is the regression line through the data. The slope of the regression line through the data defines the accuracy of the method, which is 1.05 for the lab2 and 0.84 for the lab4. The two points for each genes shown correspond to the two technical replicates of the same experiment. Table I summarizes the accuracy for the experiments in the 5 different labs.

With the ILM, we determined the global transcript concentrations in each sample, and from the ratio of concentrations in the two different conditions we obtained the fold-change in concentration. Fig. 1 shows two plots of the log_2_-fold change in the concentration for the 16 selected genes from the ILM, plotted versus the log_2_-fold change concentration as measured from RT-PCR (data from Ref. [2]). Each plot refers to a single laboratory, as each laboratory performed two replicate experiments; the figure shows the log_2 _fold change for each replicate. If the ILM agreed perfectly with the RT-PCR experiments, all data points shown in Fig. 1 would lie on the diagonal. To assess the accuracy of the ILM, we use the method used by Irizarry et al [2]: we apply a linear regression line through the data, constrained to cross the origin, and then use as a quality measure the agreement with the ideal value for this slope, which is unity. For a complete comparison of all experimental data, Table 1 reports the accuracy of the ILM for the five different laboratories. A comparison with the accuracies obtained from robust multiarray analysis (RMA) is also shown. Table 2 presents a list of log_2 _fold changes as obtained from ILM, RMA and the RT-PCR data. The performance of the ILM in this test is good.

**Table 1:** Comparison accuracies ILM and RMA

Lab.	Accuracy ILM	Accuracy RMA	rmse ILM	Offset ILM (p-value)
1	1.07	0.62	0.42	-0.07 (0.59)
2	1.05	0.64	0.60	-0.08 (0.49)
3	0.89	0.66	0.62	-0.23 (0.14)
4	0.84	0.59	0.62	-0.10 (0.28)
5	0.95	0.58	0.46	-0.07 (0.48)

Comparison of accuracies of the Inverse Langmuir Method and Robust Multi Array algorithm for the experimental data of Ref. [2]. The accuracy is the slope of the regression line as shown in Fig. 1, which has zero intercept. The accuracy for the RMA is taken from Ref. [2]. The fourth column reports the RMSE in the log_2 _fold changes between the ILM and RT-PCR data. As a check for possible biases in the ILM, we repeated the linear regression, this time allowing for intercepts. The resulting values for these intercepts are reported in column 5. The same column also reports the p-values on the intercept, which show these intercepts are not statistically significant.

**Table 2:** Log-fold changes of RT-PCR, ILM and RMA for lab 2 and 4

		Lab. 2				Lab. 4			
Gene	RT-PCR	ILM_1_	ILM_2_	RMA_1_	RMA_2_	ILM_1_	ILM_2_	RMA_1_	RMA_2_
PEX1	0.27	0.05	0.13	0.1	0.08	-0.48	0.39	0.01	0.12
PEX6	0.98	0.67	0.16	0.44	0.15	0.46	0.36	0.52	0.58
PEX7	0.08	-0.31	-0.19	-0.1	-0.07	-0.19	-0.44	-0.18	-0.11
PEX12	-0.12	-0.43	-0.55	-0.21	-0.14	-0.25	-0.18	-0.17	-0.14
BMP7	0	0.08	0.15	-0.05	0.07	-0.03	-0.18	-0.02	-0.06
CUL3	-0.11	0.19	0.31	0.08	0.44	0.18	0.14	0.08	0.22
FAF1	0.03	0.04	0.08	-0.17	0.04	0.02	-0.19	0.03	-0.08
MFNG	-0.12	0.32	0.06	0.15	-0.04	-0.39	-0.17	-0.29	0.05
MMP3	4.16	5.2	5.44	3.29	3.76	4.21	4.13	3.51	3.47
PLAU	2.46	3.1	3.43	1.94	2.09	2.8	2.55	2.11	1.88
EFEMP1	-1.88	-1.7	-1.82	-1.58	-1.6	-1.13	-0.99	-1.06	-1.01
COL5A2	-1.95	-1.77	-1.63	-1.06	-1.21	-1.33	-1.64	-1.15	-1.07
IL8	1.67	0.86	1.07	0.95	1.05	0.55	1.5	0.98	1.13
SEMA3F	1.34	-0.26	-0.42	0.12	0.16	-0.19	-0.1	0	0.08
ALOX5	-	-0.25	0.1	-0.16	0.2	-0.12	-0.59	-0.09	0
RNF167	0.35	0.5	-0.02	0.13	-0.06	0.31	0.02	0.13	-0.01

Table of log_2 _fold changes of the 16 genes in the lab 2 and lab 4 experiments, as obtained from RT-PCR measurements, and from Affymetrix experiments analyzed with the ILM and RMA. The sub-indices for ILM and RMA distinguish the technical replicates of the same oligonucleotide microarray experiment. The gene ALOX5 was not detected in the RT-PCR analysis, hence its ratio is undetermined (see Supplementary Material of Ref. [2]). This gene was not considered in the accuracy calculation.

To assess the effects of different labs and genes in the analysis, we used a general linear model of the type Δ = Gene + Lab + Gene : Lab where Δ is the difference between log_2 _fold changes for RT-PCR and ILM data. A similar analysis was done also for RMA data. In both cases the lab and gene-lab interaction effects are not significant. The p-values are for ILM *p*_Lab _= 0.14 and *p*_Gene:Lab _= 0.55, while for RMA *p*_Lab _= 0.87 and *p*_Gene:Lab _= 0.26. The majority of the variation in both methods is explained by the gene effect.

**Figure 2:**
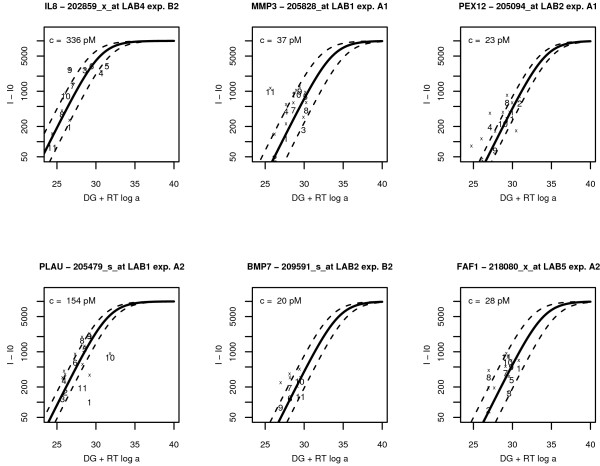
**Background-subtracted intensities vs. Langmuir model**. Background-subtracted intensities (numbers, probe numbering follows Affymetrix convention) plotted as functions of *RT *log(*K*/*K*_0_) = Δ*G *+ *RT *log *α *for six different probe sets in the experiments of Ref. [2]. As additional information we also plot the raw fluorescent intensities (crosses), so that the effect of background subtraction can be viewed by comparing the numbers with their corresponding crosses on the same *x *value. The background level is probe dependent, and was calculated using the algorithm of Ref. [13]. Typically, background subtraction has little effect on the higher intensity data, while its effect is more important for lower intensities. The lines shown are plots of Eq. (2) for three different values of the concentrations. A perfect agreement with the ILM would imply the alignment of all background-subtracted intensities (numbers) along one single line which would identify a single concentration for the whole probe set. Apart from few outliers the background-subtracted intensities (numbers) follow well the prediction of the Langmuir model (the raw fluorescent intensities (crosses) are not expected to follow the model). The solid line corresponds to the median value of the concentration, whose value, in picomolar (see text), is reported in the upper left corner of each graph. The two dashed lines are obtained from the median absolute deviation of the logarithm of the concentrations and measure the dispersion in the values of the concentrations within each probe set.

We also performed a more detailed investigation of the performance of individual probes. Fig. 2 shows the background-subtracted intensities for some of the 16 genes for which RT-PCR measurements of expression levels exist. Data are plotted as function of *RT *log(*K*/*K*_0_) = Δ*G *+ *RT *log *α*, which is calculated from Eq. (3). A perfect agreement of the data with Eq. (2) would imply their alignment along a single line, associated to a single value of the concentration *c*. In reality some spreading is always observed. The amount of spreading is linked to the accuracy of the determination of the transcript concentration in solution. The central line in the plots corresponds to the median value of the concentration, while the dashed lines are calculated from the median absolute deviation of the logarithm of the individual concentrations. Further details on the transcript concentration for each individual probe are given in Table 3 which presents few examples. The median of all concentrations per probe set and the median absolute deviation (m.a.d.) calculated from the logarithm of the concentrations are also given. Typically, only few probes deviate strongly from the median concentration, as for instance is the case for probe 9 in probe set 202859_x_at, probe 11 in probe set 205828_at, and probe 1, 10, 11 in probe set 205479_s_at. A narrow spreading (low m.a.d.) is a signature of an accurate and consistent determination of the concentrations. Besides giving an estimate of the concentration, the ILM provides also quality control of the performance of each individual probe in the probe set. For instance, probe 11 in the probe set 205828_at gives a systematically higher intensity than what is expected from the Langmuir model; this is probably due to cross-hybridization.

**Table 3:** ILM concentrations (expression levels) for each individual probe in a probe set

Probe n.	PEX12 205094_at lab2 A1	MMP3 205828_at lab1 A1	PLAU 205479_s_at lab1 A2	PLAU 205479_s_at lab4 B1
1	19	29	9	21
2	14	35	131	374
3	0	9	113	46
4	76	106	229	958
5	30	38	154	383
6	36	0	215	441
7	1	50	143	245
8	51	18	325	734
9	15	81	202	537
10	27	86	11	12
11	17	1084	28	135

median	23	38	143	374
m.a.d.	0.67	1.14	0.61	1

Examples of individual concentrations (in picomolar, see text) for each of the 11 probes of three given probe sets used in the study of Ref. [2]. The gene symbol, probe set number and laboratory number, following Ref. [2] are given. Each laboratory performed two replicate experiments (labeled as A and B) for two different mRNA samples (labeled as 1 and 2). The median value and the median absolute deviation (m.a.d.) of the logarithm of the concentrations are also given.

The absolute concentrations in Fig. 2 and Table 3 are given in picomolar (pM). The estimated values of the concentration depend in the ILM on some parameters, see Ref. [9] and Methods. In the present work we have taken these parameters from Ref. [9], where they were fitted against Affymetrix spike-in data. Hence, no adjustment of the parameters is done in the present analysis. However the absolute picomolar values should be interpreted with some care. As hybridization conditions may vary from experiment to experiment, the parameters from Ref. [9] may not be fully adequate for the present experiment. A retuning of the parameters should mainly affect the absolute concentration estimates, but not the fold-changes. The agreement between fold-changes of ILM and RT-PCR data indicates that this is plausible.

**Figure 3:**
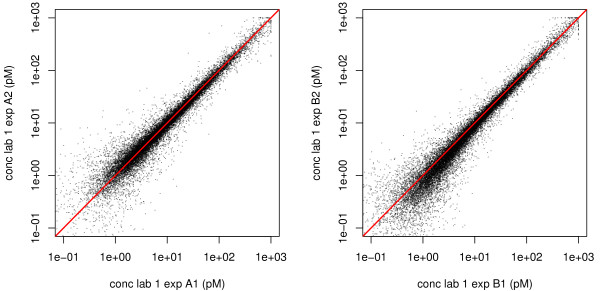
**Concentrations vs. Concentration plots of two experiments**. Examples of correlation plots of concentration vs. concentration as estimated from the ILM in two replicate experiments of laboratory 2 and laboratory 3. The red line is the diagonal. The data show very good correlation, except for the very low concentration regime in the second example. However, concentrations of about 1 pM are just above the background level somewhat below the safe detection limit, which should be placed at about 10 pM.

To evaluate to what extent slight variations in the experimental handling of the microarrays causes fluctuations in the ILM estimates of the transcript concentration, we present in Fig. 3 a plot of concentration vs. concentration of two replicate experiments performed in the same laboratory; in the left and right panels, laboratory 2 and 3, respectively. Concentrations are calculated from the median of each probe set of Eq. (4). The diagonal line is also traced. In both cases a good degree of correlation is found (in both cases the correlation coefficient is 0.97). The degree of correlation between replicate experiments is an indicator of good quality of the experiments. We note that in Fig. 3 (right) a slight unbalance is observed at very low concentrations (≈ 1 pM), which are typically below the biologically relevant range of concentrations.

## Conclusion

Most of the current algorithms for oligonucleotide microarray data analysis rely on purely statistical methods and use complex transformations for normalization, background subtraction and summarization [3]. Due to its importance in many biotechnological applications, DNA hybridization when both strands are free in solution has been widely investigated during the past decades (see e.g. [10-12]). It is therefore natural to seek for an application of the insights obtained during those studies to the case of hybridization in DNA microarrays. The advantages of microarray data analysis algorithms based on physico-chemical properties of the underlying hybridization process were indeed emphasized in few studies [4-6].

Here we presented the Inverse Langmuir Method, a new algorithm for Affymetrix Genechips data analysis which is based upon DNA and RNA hybridization thermodynamics. The ILM consists of two steps: the background subtraction and the transcript concentration estimation for the background-subtracted intensities. These two steps, which were accurately fine-tuned and tested separately on Affymetrix spike-in data in previous publications [9,13], are here combined into a single algorithm.

To validate the ILM, we applied it to publicly available microarray data from a multi-lab comparison study [2] in which microarray experiments were performed on mRNA samples where different expression levels of few genes are induced. The log_2 _fold change between these two samples, as obtained from RT-PCR experiments, agrees well with the log_2 _fold change as obtained with the ILM, indicating that the ILM determines changes in the expression level accurately. A comparison of replicated experiments shows that the ILM is not very sensitive to slight variations in the experimental handling of the microarrays, and hence gives a very good degree of reproducibility. Since the ILM yields independent concentration estimates per probe, it allows for the identification of outlier probes. Differently from other physico-chemical inspired algorithms [5,6] the ILM uses a much smaller number of adjustable parameters. This is because the hybridization free energies Δ*G *and Δ*G' *entering in Eq. (3) are obtained from RNA/DNA and RNA/RNA tabulated stacking parameters obtained from melting experiments in solution [10,11].

In the near future, we plan to make a freely available version of the ILM available through .

## Methods

In general, in a microarray experiment, the intensity measured from a given probe with sequence *s *can be decomposed into two contributions

(1) *I*(*c*) = *I*_sp_(*c*) + *I*_0_.

The first term, *I*_sp_, is the specific part of the signal, which depends on the transcript concentration in solution *c*, which is the gene expression level one needs to estimate. The second term, *I*_0_, is due to cross-hybridization or other spurious effects. This is the amount of fluorescent signal one measures even in absence of the specific transcript (*c *= 0); it is sequence-dependent, since some probes (for instance CG-rich ones) are more prone to cross-hybridize than others. Equation (1) neglects competitive effects between specific and non-specific hybridization (see e.g. [6]), which arise if the intensity approaches saturation. Since typically the background intensity *I*_0 _is only a few percent of the saturation intensity, this effect only plays a significant role if the total intensity is dominated by the contribution of specific hybridization. In that case, however, the resulting overestimation of *I*_0 _does not affect the main purpose of the ILM, which is to estimate transcript concentrations.

In the ILM, the estimate of *I*_0 _is obtained as described in Ref. [13]. This background estimation scheme combines information from the intensities of nearby probes with a purely sequence-based estimation of the affinity for non-specific binding. The latter correlates with the stacking parameters obtained from melting experiments in solution [10]. The performance of this background estimation scheme has been extensively tested [13], with excellent results, on pure background data from Affymetrix spike-in experiments.

Once the background is subtracted, the transcript concentration is estimated through the Langmuir adsorption model. This model links the fluorescent intensity to the transcript concentration:

(2) $$I_{\text{sp}}(c)=\frac{AcK}{1+cK}.$$

Here *A *is the saturation value, i.e. the limiting value of intensity reached at large concentrations. At saturation all probes are hybridized and the microarray is no longer sensitive to a further increase in transcript concentration. An analysis of Genechips intensity histograms [14] of several organisms reveals that there is a sharp drop at intensities in the range 10,000 ≲ *I *≲ 15,000, and very few probes have a higher intensity. This suggests that *A *varies within this range. Recent literature [15-17] reported significant variations in *A *from probe to probe, attributed to the post-hybridization washing step. Lacking a detailed quantitative understanding of the washing process, we take a fixed value of *A *= 10,000, as done in Ref. [9].

Extensive tests on Affymetrix spike-in experiments [9] showed that most likely two competing types of chemical reactions contribute to the *I*_sp_: 1) The hybridization of the transcript sequence to the complementary probe sequence. This reaction is characterized by a hybridization free energy Δ*G*, which is sequence dependent (for instance, CG-rich transcript fragments bind more strongly to their complementary probes). 2) Hybridization in solution of the transcript with partially complementary fragments from other transcripts, characterized by a second hybridization free energy Δ*G'*. This reaction leads to an effective reduction of the target concentration by a factor *α *≤ 1. Combining the effects of 1) and 2) one gets

(3) $$K/K_{0}=\alpha \left(\frac{\Delta G^{\prime }}{RT^{\prime }},\tilde{c}\right)\exp(\Delta G/RT)=\frac{\exp(\Delta G/RT)}{1+\tilde{c}K_{0}\exp(\Delta G^{\prime }/RT^{\prime })}$$

with *R *the gas constant (= 1.99 cal/(mol·K)) and *K*_0 _is a unit constant with a dimension of inverse concentration. The hybridization free energies Δ*G *for transcript-probe hybridization are calculated from the nearest neighbor model, with tabulated experimental data for DNA/RNA [10] duplex formation in solution. Recent test experiments [18] on spotted arrays showed a strong correlation between microarray fluorescent intensities and hybridization free energies from stacking parameters in solution, corroborating our underlying assumption that hybridization in solution is very similar to hybridization on a microarray. The hybridization free energies Δ*G' *enter in the factor *α *and are associated to transcript-transcript hybridization in solution as well as self-folding; these are also calculated from the nearest neighbor model, with tabulated experimental data for RNA/RNA [11] duplex formation in solution. Both types of these hybridization free energies do not contain fitting parameters. The ILM uses therefore only four global adjustable parameters which are *A*, *T*, *T' *and $\tilde{c}$, with values as obtained by regression analysis of Affymetrix spike-in data [9]. In general, one may wonder whether different experimental circumstances require a refitting of these parameters. For instance, the parameter *α *describes hybridization between partially complementary transcripts in solution. Two different experiments with, say, human mRNAs extracted from two different tissues contain different sequences in solution and hence *α *could in principle be different. Also, a significant decrease in total concentration, for instance, will impact the balance between the two competing types of processes [19], and would result in a reduced $\tilde{c}$. In a previous publication [20] it has been shown that the model of Eqs. (2,3) describes well spike-in HGU133 data, with the same parameters *A*, *T*, *T' *and $\tilde{c}$ as fitted on HGU95 [9]. We expect thus that a refitting does not significantly improve the results. Indeed, the good agreement with the RT-PCR data presented in this paper is an indication that the values of parameters as fitted Ref. [9] are appropriate for the study presented here. Note the competing effect of the two coupled chemical reactions: an increase of the affinity of the transcript-probe hybridization (increase of Δ*G*) leads to an overall increase of the fluorescence signal. The effect is opposite for an increase in the affinity of transcript binding in solution (higher Δ*G'*), as this leads to a decrease of the signal. We also note that Eq. (3) defines an *extended *Langmuir model in which *K *can be viewed as a measurements of the *effective *affinity for binding of the transcript to the probe sequence, in which the effect of reaction 2) is incorporated.

A given probe sequence uniquely determines *K*, hence the transcript concentration can be found by inverting Eq. (2) as

(4) $$c=\frac{1}{K}\frac{I_{\text{sp}}}{A-I_{\text{sp}}}.$$

This equation forms the basis of the Inverse Langmuir Method (ILM). For a probe set containing *n *probe sequences, Eq. (3) yields *n *values of *K *and from Eq. (4) one gets *n *independent estimates of the transcript concentration. We take the median estimate as our best estimate for the value of the transcript concentration.

In summary, the ILM measures the expression level (transcript concentration) as follows. The first step in the analysis is the background estimation [13] and its subtraction from the raw intensities to yield *I*_*sp *_= *I *- *I*_0_. Next, using Eqs. (3) and (4) one obtains a value of the concentration per probe. The median concentration within the probe set is then taken as global transcript concentration.

## Authors' contributions

GB and EC planned and supervised the research and wrote the paper. GM wrote the code for data analysis and analyzed the experimental data.
